# The Impact of Tube Type, Centrifugation Conditions, and Hemolysis on Plasma Circulating MicroRNAs

**DOI:** 10.3390/diagnostics14212369

**Published:** 2024-10-24

**Authors:** Belén Pastor-Navarro, Marta Ramírez-Calvo, Isabel Gil Aldea, Isabel Cortell Granero, José A. López Guerrero

**Affiliations:** 1 Laboratorio de Biología Molecular, Fundación Instituto Valenciano de Oncología, 46009 Valencia, Spain; bpastor@fivo.org (B.P.-N.); mramirezc@fivo.org (M.R.-C.); igaldea@yahoo.es (I.G.-A.); icortell@fivo.org (I.C.-G.); 2 Peptide and Protein Laboratory, Department of Medicinal Chemistry, Unidad Mixta Centro de Investigación Príncipe Felipe, 46012 Valencia, Spain; 3 Medicine and Health Sciences Faculty, Universidad Católica de Valencia San Vicente Mártir, 46001 Valencia, Spain

**Keywords:** microRNAs, preanalytical conditions, SPREC code

## Abstract

Background: In recent years, liquid biopsy has emerged as a promising tool for the diagnosis and prognosis of numerous diseases, including cancer. Among the biomolecules analyzed in liquid biopsies are plasma circulating microRNAs (miRNAs), small non-coding RNAs that have proven to be crucial in the regulation of gene expression and the pathobiology of different health conditions, making them useful as biomarkers. However, variations in preanalytical conditions during biospecimen collection and processing can affect the analytical results. Objectives: Herein, we determined how the type of collection tube, the number of centrifugations, and the degree of hemolysis can affect plasma circulating miRNA levels. Methods: A cohort of 11 healthy donors was included. Whole blood was collected and handled in three different conditions, and miRNAs levels were analyzed using quantitative RT-PCR. Results: Our results show that the differences in the type of preservative tubes influence hemolysis, measured as OD at 414 nm. Moreover, the number of centrifugations performed also altered miRNAs levels, increasing or decreasing them depending on the miRNA analyzed. Hence, our study shows that alterations in preanalytical conditions affect miRNAs levels, particularly the number of centrifugations and the type of collection tubes. Conclusions: In our work, we highlight the importance of registering the preanalytical conditions in a standardized way that might be considered when analytical results are obtained.

## 1. Introduction

Tissue biopsies remain the gold standard for cancer diagnosis; however, they are associated with numerous limitations, including invasiveness, sampling bias, limited repeatability, high time and cost demands, and potential complications and contraindications. In contrast, liquid biopsies have emerged as a promising complementary tool for cancer diagnosis and management. Consequently, there has been a notable shift in oncology research toward liquid biopsies in recent years. These techniques involve the detection of cancer-derived components released into body biofluids, effectively addressing many of the inherent disadvantages associated with traditional tissue biopsies [1,2].

Among these components are circulating tumor cells; circulating tumor DNA, RNA, and microRNAs (miRNAs or miRs); and extracellular vesicles. These elements provide comprehensive genomic, epigenetic, transcriptomic, and proteomic information regarding tumors, metastatic sites, and tumor progression [1,2,3,4].

MiRNAs are a family of 21- to 22-nucleotide long, small non-coding RNAs found in eukaryotic cells, that are considered one of the main post-transcriptional regulators of gene expression [5,6]. A dysregulation of miRNA expression levels in circulation is related with some diseases, like cancer [7,8]. These small molecules can be found in a variety of body fluids, such as blood, urine, or semen, and are extremely stable due to their encapsulation into microvesicles and their association with protein complexes such as argonaute 2 and lipoproteins [9]. Thus, all these features make circulating miRNAs a potential non-invasive tool for diagnosis and prognosis of cancer [10,11].

However, the optimal preanalytical conditions for analyzing blood miRNAs remain unclear. This includes determining the appropriate collection tubes, acceptable hemolysis levels in samples [12,13,14], and the ideal centrifugation protocols to be implemented [15,16,17].

The choice of collection tubes, influenced by variations in anticoagulant reagents, has been shown to affect miRNA quantification [16,17,18,19]. Additionally, both the number and speed of centrifugation steps can impact miRNA levels, as it has been demonstrated that low expressed miRNAs can be affected by a second high-speed centrifugation step [17].

Regarding hemolysis, some studies have shown that, during blood collection or sample processing, red blood cells rupturing can arise, having an impact of levels of certain plasma and serum miRNAs. In particular, the expression levels of hsa-miR-16 (miR-16) have been shown to be higher in hemolyzed samples compared to non-hemolyzed ones, measuring hemolysis as optical density (OD) at 414 nm [12,13,14,20]. However, implementing a cut-off value of <0.2 for hemolysis has been suggested to significantly reduce variability in miRNA quantification across sample series [14].

Hence, due to the high number of issues that can affect miRNAs quantification, it becomes crucial to study preanalytical conditions that could alter the final characterization of the biomolecules of interest, as there are no existing standard operating procedures (SOPs). To address this gap, in 2009 the International Society for Biological and Environmental Repositories developed the Standard PReanalytical Code (SPREC) [21]. This comprehensive tool considers all preanalytical conditions, aiming to report the key variables that may impact analytical results.

In the present study, we compared four miRNAs implicated in the pathobiology of prostate cancer (PCa) [8] across different blood collection tubes, with different centrifugation protocols, to assess how these conditions influence miRNAs analysis. Additionally, we evaluated the correlation between hemolysis and miR-16 levels within our sample series.

## 2. Methods and Materials

### 2.1. Ethics Statement

This study received approval from the Clinical Research Ethics Committee of the Fundación Instituto Valenciano de Oncología (FIVO) during the meeting held on 18 December 2021 (LBM-01-20 CECILIA). Written informed consent was obtained from all participants prior to sample collection.

### 2.2. Subjects’ Characteristics

Whole blood from 11 male volunteers with no history of cancer or other chronical disease prior the blood draw was collected. The median age was 46 years, ranging from 39 to 59 years.

### 2.3. Blood Collection and Plasma Isolation

Cell-Free DNA BCT tubes (Streck Inc., La Vista, NE, USA) and S-Monovette^®^ EDTA K3 (Sarstedt AG & Co., Nümbrecht, Germany) were used to collect seven milliliters of peripheral blood from all men. Then, plasma was separated according to FIVO Biobank standard procedures, and all the preanalytical information is summarized in Figure S1. SPREC codification was used for reporting preanalytical conditions [21].

Specifically, EDTA tubes were stored at 4 °C and processed 30 min after blood collection. First, EDTA tubes were centrifuged at 1600× *g* for 10 min at room temperature (RT). Then, supernatant was transferred to 1.5 mL tubes and centrifuged at 16,000× *g* for 10 min at 4 °C. Finally, the supernatant was recovered and stored at −80 °C.

Regarding STRECK (ST) tubes, they were stored at RT and protected from light for 4 h. Then, tubes were centrifuged at 1600× *g* for 10 min at RT. After centrifugation, half of the supernatant was stored at −80 °C as the ST-1 condition. The remain supernatant was transferred to 1.5 mL tubes and centrifuged at 16,000× *g* for 10 min at 4 °C. Finally, supernatant was recovered and stored at −80 °C as the ST-2 condition.

According to the objective, plasma aliquots from ST tubes were stored both after the first (ST-1) and second (ST-2) centrifugation steps (Figure S1). An average of three plasma aliquots from all conditions were stored at −80 °C FIVO Biobank in 1.5 mL aliquots until use.

### 2.4. RNA Isolation

Plasma aliquots were thawed at room temperature for 15 min with shaking. Subsequently, 200 μL of plasma were used to isolate the total RNA using the miRNeasy Serum/Plasma Kit (Qiagen N.V., Hilden, Germany) in accordance with the manufacturer’s protocol.

The OD of plasma samples before RNA isolation and the concentration of total RNA in each sample were taken using a NanoDrop 1000 spectrophotometer (Thermo Scientific, Wilmington, DE, USA) at wavelengths of 414 nm and 260 nm, respectively.

### 2.5. Reverse Transcription PCR (RT-PCR)

Prior to RT-PCR, a RT 0.05× working solution was prepared by pooling 5x miRNAs primers from hsa-miR-16 (ID: 000391), hsa-miR-21 (ID: 000397), hsa-miRNA-182 (ID: 002334), hsa-miR-125b (ID: 000449), and hsa-miR-375 (ID: 00564) (TaqMan MicroRNA Assays, Applied Biosystems, Thermo Fisher Scientific, MA, USA) using Tris-EDTA (TE) buffer 1×.

Then, the RT reaction was performed using the TaqMan MicroRNA Reverse Transcription kit (Applied Biosystems, Thermo Fisher Scientific, MA, USA) by adding 150 ng of total RNA in a total volume reaction of 15 μL, which included 2 mM dNTPs, 3.3 U/mL MultiScribe reverse transcriptase, 1× reverse transcription buffer, 0.25 U/mL RNase inhibitor, and primer pool 0.05×. The reaction was performed using the Veriti Pro Thermal Cycler (Applied Biosystems, Thermo Fisher Scientific, MA, USA) at 16 °C for 30 min, 42 °C for 30 min, and 85 °C for 5 min.

### 2.6. cDNA Pre-Amplification

Similar to the process used for the RT primer pool, a pre-amplification 0.2× working solution was prepared by pooling 20× miRNAs primers from miR-16, miR-21, miRNA-182, miR-125b, and miR-375 (TaqMan MicroRNA Assays, Applied Biosystems, Thermo Fisher Scientific, MA, USA) using TE buffer 1×.

Subsequently, 5 μL of cDNA were pre-amplified using the TaqMan PreAmp Master Mix (2×) according to the manufacturer’s instructions (Applied Biosystems, Thermo Fisher Scientific, MA, USA). For this reaction, 2.5 μL of cDNA sample was added to a total volume of 12.5 μL containing 6.25 μL of TaqMan PreAmp Master Mix (2×), 3.625 μL of pooled assay mix (0.2×), and nuclease-free water. The reaction was performed using a Veriti Pro Thermal Cycler (Applied Biosystems, Thermo Fisher Scientific, MA, USA) for 14 cycles at 95 °C for 10 min, 95 °C for 15 s, and 60 °C for 4 min.

### 2.7. Quantitative Real-Time PCR (qRT-PCR)

A 1:10 dilution of the preamplified cDNA in TE buffer 1× was performed prior to quantification. cDNA was added to a final reaction volume of 10 μL, which contained 20× TaqMan MicroRNA assay primers (Applied Biosystems, Thermo Fisher Scientific, MA, USA), TaqMan Gene Expression Master Mix (2×) (Applied Biosystems, Thermo Fisher Scientific, MA, USA), and nuclease-free water. The reaction was performed using a LightCycler 480 (Roche, Basel, Switzerland) at 95 °C for 10 min and 40 cycles at 95 °C for 15 s and 60 °C for 60 s. Technical triplicates of each condition were performed.

### 2.8. Statistical Analysis

The Cq values, with Cq referring to quantification cycle, obtained from qRT-PCR provide a quantitative measure of the target miRNA, with lower Cq values indicating higher concentrations of the target miRNA. Since Cq values represent a nonlinear concept, it is not advisable to use the original Cq values for statistical analysis. Therefore, using miR-16 as a reference miRNA, the relative levels of each miRNA were calculated using the equation 2^−ΔCq^, where ΔCq = mean Cq(miRNA)−mean Cq(miR-16) [22].

Normality was assessed in each study population using the Kolmogorov–Smirnov test. Quantile –Quantile plots were generated for each analyzed population (Table S1 and Figures S2–S4). Outliers were identified using the ROUT method with a Q value of 1%.

The raw Cq values of miRNAs among the different conditions were analyzed using ordinary one-way parametric ANOVA and Tukey’s multiple comparisons test. The relative levels of miRNAs among different conditions, as previously defined, were compared using the non-parametric Kruskal–Wallis test followed by Dunn’s multiple comparisons test.

The correlations between the variables were assessed using the non-parametric Spearman correlation coefficient.

All analyses were performed by GraphPad Prism (version 10.0; GraphPad Software).

## 3. Results

### 3.1. MiRNAs Levels Among Different Conditions and Their Correlations

**Figure 1:**
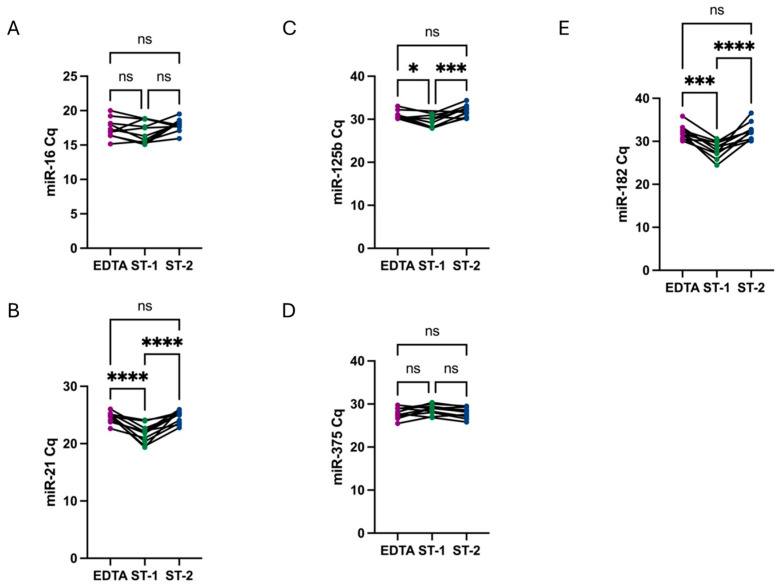
Comparison of miR-16 (**A**), miR-21 (**B**), miR-125b (**C**), miR-375 (**D**), and miR-182 (**E**) Cq values among conditions. No significant differences in miR-16 levels were observed across different conditions. Regarding PCa miRNAs, in all cases, Cq values were lower in ST-1 samples (green), with a single centrifugation step, indicating a higher miRNAs quantity. However, when comparing EDTA tubes (purple) and ST-2 (blue), both with a second high-speed centrifugation step, there was no significant differences between them. Conversely, in the case of miR-375, Cq values were higher in ST-1 samples, although without statistical significance. Stars represent level of significance with (****) for *p*-value < 0.0001, (***) for *p*-value 0.0002, (*) for *p*-value 0.03, and ns for *p*-value > 0.05.

The levels of all analyzed PCa miRNAs were compared across different conditions using the raw Cq data obtained from qRT-PCR analysis. These results indicated that, except for miR-375, all miRNA levels were higher in ST-1 samples, presenting as lower Cq values. However, when comparing EDTA tubes and ST-2 samples, both subjected to a secondary high-speed centrifugation step, no significant differences were observed between the two groups (Figure 1). Notably, in the case of miR-375, although the difference was not statistically significant, its levels were lower in ST-1 samples.

**Figure 2:**
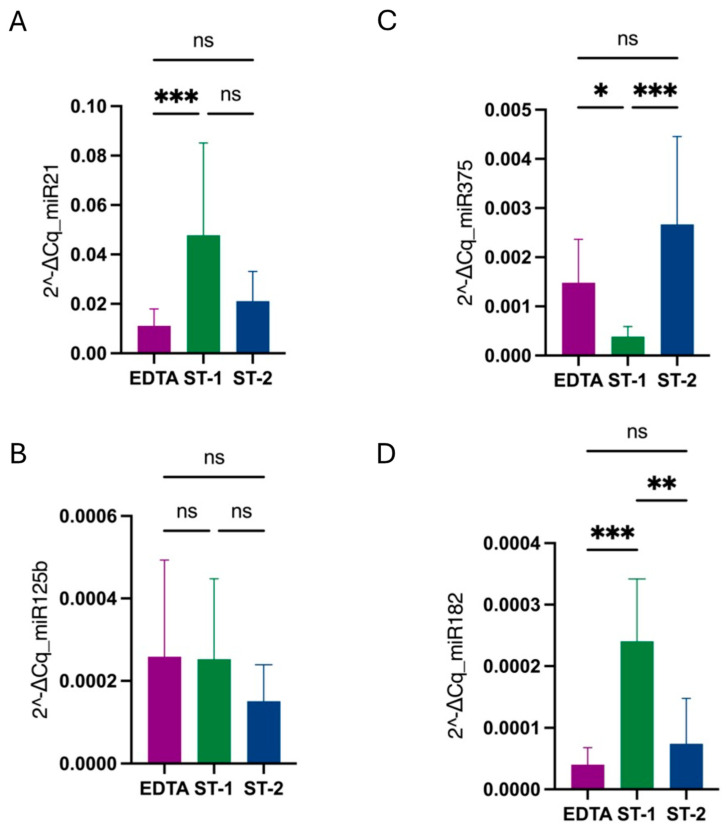
Comparison of miR-21 (**A**), miR-125b (**B**), miR-375 (**C**), and miR-182 (**D**) levels among conditions when normalized to the miR-16 reference gene using 2^−ΔCq^ values. In the case of miR-21 and miR-182, levels were higher in ST-1 (green) compared with the other conditions, with no differences between EDTA (purple) and ST-2 (blue). For miR-375, the ST-1 group was the one with lower levels, while for miR-125b, there were no differences among groups. Stars represent level of significance with (***) for *p*-value 0.0002, (**) for *p*-value 0.002, (*) for *p*-value 0.03, and ns for *p*-value > 0.05.

Additionally, when normalizing the levels of each miRNA using miR-16 as a reference gene, miR-21 and miR-182 levels were higher in the ST-1 condition compared to the other conditions, with no significant differences between EDTA and ST-2. For miR-375, the ST-1 group exhibited the lowest levels, whereas no differences were observed among groups for miR-125b (Figure 2).

These findings suggest that miRNA levels are influenced not by the type of collection tube, but by the number and speed of centrifugations during sample processing.

### 3.2. Correlation of MiRNAs Among Different Conditions

As shown in Figures S5–S7, no correlation was observed across the different conditions analyzed for each individual miRNA. Therefore, based on these data, there are no consistent results that allow for the identification of optimal conditions due to the lack of intrasample correlation.

### 3.3. OD Levels Among Different Conditions

**Figure 3:**
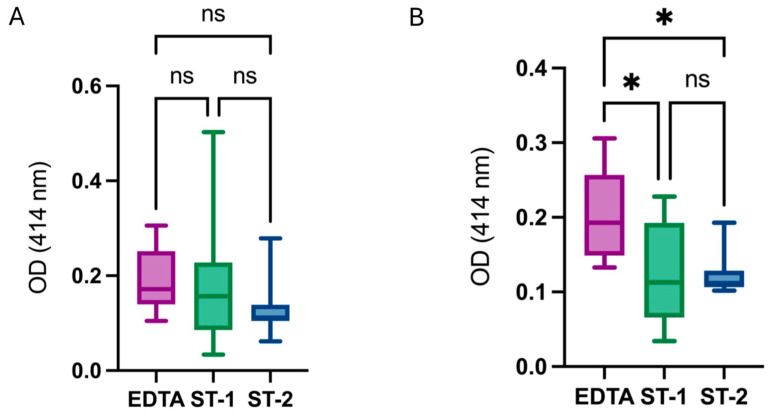
Comparison of OD values measured at 414 nm among the different conditions. (**A**) OD values among groups including all samples analyzed. (**B**) OD values among groups removing outlier values. Hemolysis, measured as OD at 414 nm, was higher in EDTA tubes compared to ST tubes. Stars represent level of significance with (*) for *p*-value < 0.03, and ns for *p*-value > 0.05.

When comparing the three conditions, no significant differences in OD were observed among them (Figure 3A). However, after removing outliers, OD was significantly higher in EDTA tubes, while no differences were detected between samples collected in ST tubes following one or two centrifugation steps (Figure 3B). The outliers corresponded to two subjects, where both ST-1 and ST-2 conditions were excluded due to issues during blood collection that resulted in marked hemolysis, thus biasing the group results.

These findings suggest that EDTA tubes are more susceptible to hemolysis, as the OD value was significantly higher compared to ST tubes.

### 3.4. MiRNAs Levels Among Different Conditions and Their Correlations with Hemolysis

MiR-16 levels are generally expected to remain constant across different samples, as it is considered a reference gene [23,24]. However, certain conditions, such as hemolysis or variations in centrifugation steps and speeds, may affect its levels. To investigate this, we compared miR-16 levels across different collection tubes and centrifugation steps in the case of ST tubes.

Our analysis revealed no significant differences in miR-16 levels between EDTA and ST tubes, regardless of the number of centrifugation steps performed in the latter (Figure 1).

**Figure 4:**
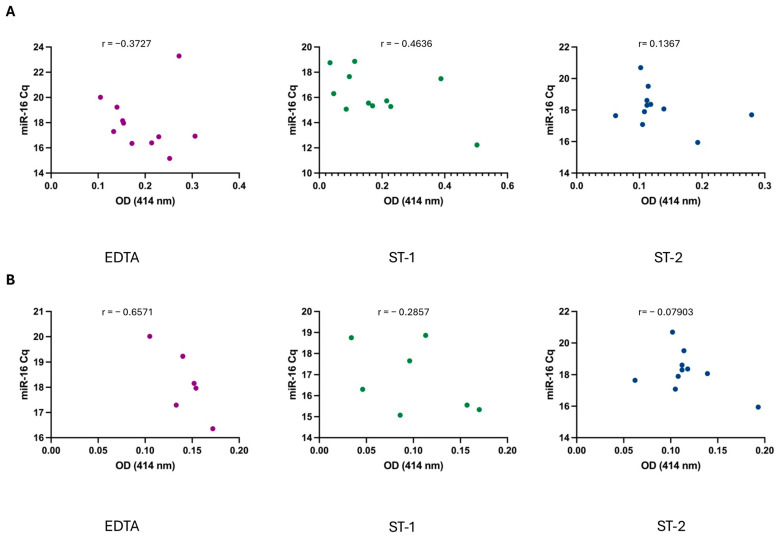
(**A**) Correlation between sample hemolysis, measured as OD at 414 nm, and miR-16 Cq values across the different conditions. (**B**) Correlation when applying a cut-off <0.2. Correlation was analyzed using non-parametric Spearman correlation test. *p*-values were not significant for any condition when alpha = 0.05. r = Spearman r.

Following the analysis of OD and miR-16 levels, a correlation analysis was conducted for each sample. No significant correlation was observed between these values under any of the three conditions tested (Figure 4A), indicating that there is no direct relationship between hemolysis, as measured by OD, and miR-16 levels. Moreover, several studies have recommended implementing a cut-off value of <0.2 absorbance units at 414 nm to effectively exclude hemolyzed samples. Based on this criterion, we reanalyzed the previous data. While this adjustment improved the correlation under the EDTA condition (Figure 4B), a strong correlation between hemolysis and miR-16 levels was still not observed.

**Figure 5:**
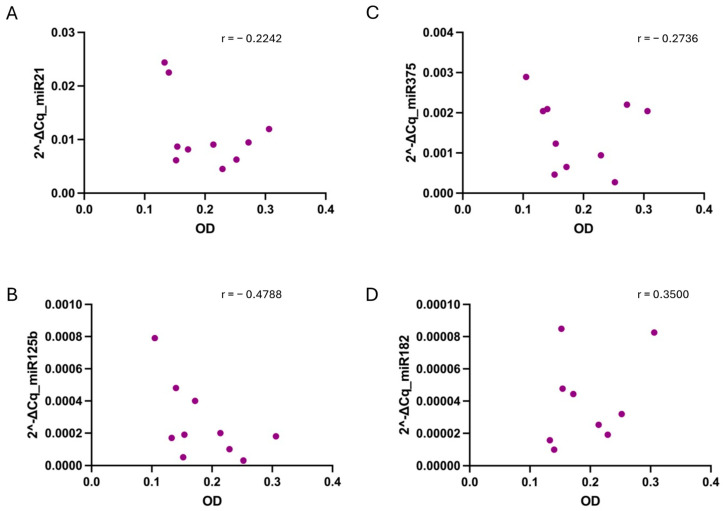
Correlation between sample hemolysis, measured as OD at 414 nm, and miR-21 (**A**), miR-125b (**B**), miR-375 (**C**), and miR-182 (**D**) relative values determined in collected in EDTA tubes. No strong correlation, tested using the non-parametric Spearman correlation test, was found for any of the cases. *p*-values were not significant for any condition when alpha = 0.05. r = Spearman r.

**Figure 6:**
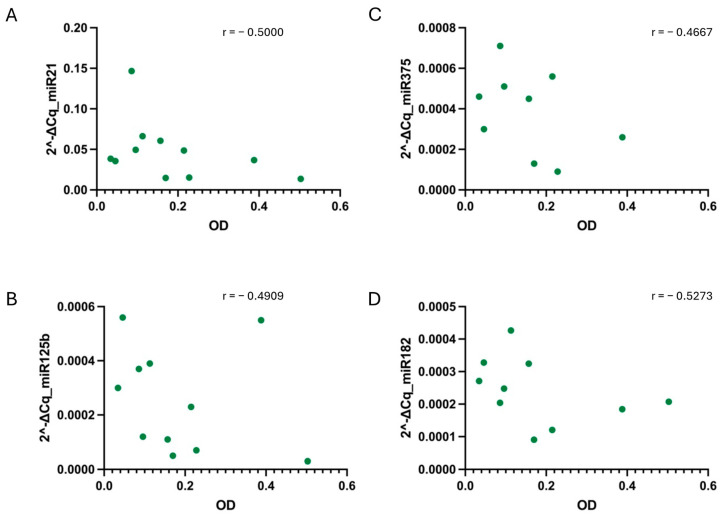
Correlation between sample hemolysis, measured as OD at 414 nm, and miR-21 (**A**), miR-125b (**B**), miR-375 (**C**), and miR-182 (**D**) relative values determined under the ST-1 condition. No strong correlation, tested using the non-parametric Spearman correlation test, was found for any of the cases. *p*-values were not significant for any condition when alpha = 0.05. r = Spearman r.

**Figure 7:**
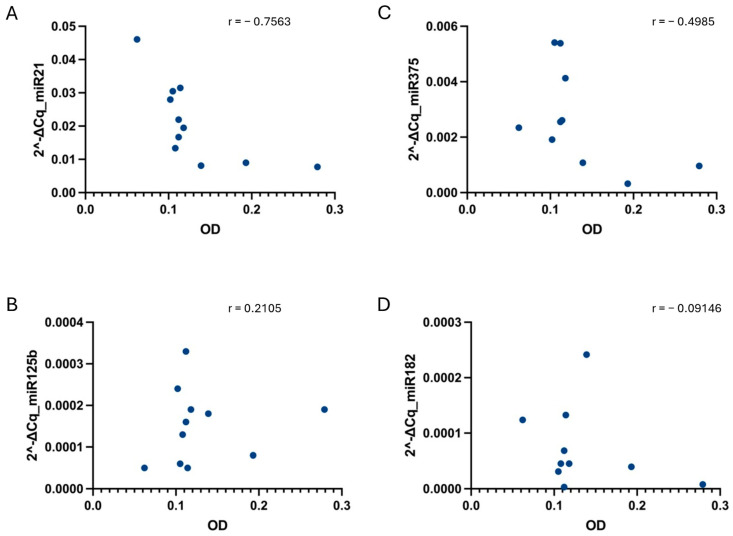
Correlation between sample hemolysis, measured as OD at 414 nm, and miR-21 (**A**), miR-125b (**B**), miR-375 (**C**), and miR-182 (**D**) relative values determined under the ST-2 condition. No strong correlation, tested using the non-parametric Spearman correlation test, was found for any of the cases. *p*-values were not significant for any of the conditions when alpha = 0.05. r = Spearman r.

Furthermore, when correlating the relative PCa miRNAs levels and hemolysis, no strong correlation was found in any of the conditions (Figure 5, Figure 6 and Figure 7).

## 4. Discussion

Circulating miRNAs are present in various body fluids and have garnered significant interest in recent years due to their potential as disease biomarkers [25,26,27]. Research has predominantly focused on plasma and serum, as these fluids are easily accessible, can be collected with minimal risk or discomfort, and are routinely obtained during clinical evaluations. Consequently, numerous studies have identified novel miRNA biomarkers in plasma and serum, leading to a growing need for standardized and detailed reporting methods.

The type of collection tube is a critical factor to consider when analyzing blood miRNAs, as it has been reported that the choice of anticoagulant influences not only preanalytical conditions, such as pre-centrifugation delay and temperature, but also miRNA levels [16,19]. Although EDTA tubes are widely used in both research and diagnostic procedures due to their low cost, ST tubes are increasingly gaining popularity in clinical settings. This shift is attributed to the practical advantages of ST tubes, including their ability to tolerate longer pre-centrifugation delays and the absence of a requirement for cold storage [17].

Moreover, it has been reported that miRNA recovery is influenced not only by the type of collection tube but also by the number and speed of centrifugation steps during sample processing. A secondary centrifugation step is generally recommended, with the notable exception of miRNAs exhibiting low levels of expression [17].

Furthermore, the initial reports on the effects of hemolysis during blood collection and processing, published in 2011, suggested that hemolysis can significantly affect the levels of specific miRNAs in hemolyzed samples, particularly miR-92a, a potential biomarker in various cancers [14]. Hemolysis is an important factor to consider in miRNA studies. Additionally, several studies have provided strong evidence that the levels of miR-16, commonly used as reference gene, are associated with hemolysis, as determined by spectrophotometric measurements [12,13,20]. However, our results show no correlation between miR-16 levels and hemolysis across the different conditions, even after applying a cut-off of <0.2 (Figure 4). These findings suggest that, although miRNAs may be associated with red blood cells, there is no direct correlation between hemolysis and altered miR-16 levels, supporting its use as an accepted control in miRNA analysis.

Furthermore, as shown in Figure 3, differences in the type of preservative between EDTA and ST tubes significantly influence hemolysis, measured as OD at 414 nm. Our results indicate that EDTA tubes are more prone to hemolysis, displaying significantly higher hemolysis levels compared to ST tubes, regardless of the number of centrifugations performed.

In this study, we compared miRNA levels across different collection tubes and centrifugation steps. Our results indicate that for both EDTA and ST-2 conditions, where a second high-speed centrifugation step was performed, miRNA levels were lower compared to ST-1. However, for miR-375, its levels increased following the second high-speed centrifugation, suggesting that it may be associated with other biomolecules, such as lipoproteins or exosomes, which hinder its detection and are released during high-speed centrifugation. Therefore, we demonstrate that the processing steps, rather than the type of collection tube, primarily determine the levels of the miRNAs analyzed.

Interestingly, we observed statistically significant differences in miRNA levels across different conditions when comparing groups (Figure 1 and Figure 2); however, no correlation was found within individual samples (Figures S5–S7). This may be explained by the presence of uncontrolled factors that could influence variability within groups but not between groups. Such factors may dilute the correlation within individual samples, while not necessarily affecting the differences in miRNA levels between groups.

However, a larger sample size and the inclusion of EDTA tubes processed with a single centrifugation step are required to achieve more robust results.

All these results show the importance of considering preanalytical conditions to obtain comparable results, since small variations in these steps can lead to obtaining different conclusions, as has been demonstrated in the present study. Despite the high variability among SOPs for biofluid analysis [28], it is strongly recommended to use the SPREC code to document all preanalytical variables. This approach enables the identification of samples processed under similar conditions, thereby facilitating robust and highly reproducible results.

## 5. Conclusions

In conclusion, hemolysis levels should be assessed in every single study as it could be significantly affecting the miRNAs analysis, while blood collection using ST tubes could decrease hemolysis. Additionally, while a second high-speed centrifugation step typically reduces miRNA levels, the optimal processing procedures should be tailored for each study based on its specific objectives and the miRNAs being assessed. Hence, the comparison of results among studies that do not follow the same procedure is not appropriate as a number of biases might affect the consistency of the analytical results.

## Data Availability

The original contributions presented in the study are included in the article/Supplementary Materials, and further inquiries can be directed to the corresponding author.

## Supplementary Materials

The following supporting information can be downloaded at: https://www.mdpi.com/article/10.3390/diagnostics14212369/s1, Figures S1–S7 and Table S1. Figure S1. Summary of the procedures followed and the type of samples obtained. SPREC codes were used for reporting biospecimen handling. Created with BioRender.com. BLD: Blood (whole); PED: Potassium EDTA; SCK: Nonaldehyde-based stabilizer for cell-free nucleic acids; PP: polypropylene. Figure S2. Quantile-Quantile plots using Cq values from miR-16 (A), miR-21 (B), miR-125b (C), miR-375 (D) and miR-182 (E) among conditions. Normality was assessed using Kolmogorov-Smirnov test and *p*-values were summarized on Table S1. Based on Cq values, all miRNAs follow a Gaussian distribution in all conditions, thus parametric test were used for their analysis. Figure S3. Quantile-Quantile plots using 2−ΔCq values from miR-21 (A), miR-125b (B), miR-375 (C) and miR-182 (D) among conditions. Normality was assessed using Kolmogorov-Smirnov test and *p*-values were summarized on Table S1. Based on 2−ΔCq values, not all miRNAs follow a Gaussian distribution in all conditions, thus non-parametric tests were used for their analysis. Figure S4. Quantile-Quantile plots using OD values from the three different conditions. Normality was assessed using Kolmogorov-Smirnov test and *p*-values were summarized on Table S1. Based on OD values, only ST-2 conditions did not follow a Gaussian distribution, thus non-parametric tests were used when considering this condition. Figure S5. Correlation, using 2−ΔCq values from miR-21 (A), miR-125b (B), miR-375 (C) and miR-182 (D), between EDTA and ST-1 conditions. Correlation was assessed using non-parametric Spearman tests and P-value was non-significant in any condition, when alpha=0.05. r = Spearman r. Figure S6. Correlation, using 2−ΔCq values from miR-21 (A), miR-125b (B), miR-375 (C) and miR-182 (D), between EDTA and ST-2 conditions. Correlation was assessed using non-parametric Spearman tests and *p*-values was non-significant in any condition, when alpha = 0.05. r = Spearman r. Figure S7. Correlation, using 2−ΔCq values from miR-21 (A), miR-125b (B), miR-375 (C) and miR-182 (D), between EDTA and ST-2 conditions. Correlation was assessed using non-parametric Spearman tests and *p*-values was non-significant in any condition, when alpha = 0.05. r = Spearman r. Table S1. Kolmogorov-Smirnov test results for each study population. The test is considered significant at an alpha level of 0.05.

## Abbreviations

Cq	quantification cycle
FIVO	Fundación Instituto Valenciano de Oncología
miRNAs (or miRs)	microRNAs
OD	optical density
PCa	prostate cancer
RT	room temperature
RT-PCR	reverse transcription PCR
SOPs	standard operating procedures
SPREC	Standard PReanalitical Code
STRECK	ST
TE	Tris-EDTA
