# Supplementary material for: The Impact of Tube Type, Centrifugation Conditions, and Hemolysis on Plasma Circulating MicroRNAs
Source: Diagnostics (Basel). 2024 Oct 24;14(21):2369. doi: 10.3390/diagnostics14212369 (PMC11545111; doi:10.3390/diagnostics14212369)
Supplement: Supplementary file 1 [file diagnostics-14-02369-s001.zip › Supplementary figures.pdf]

	Type of sample	Type of primary container	Pre-centrifugation	Centrifugation 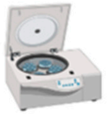	Second centrifugation 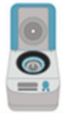	Post-centrifugation delay	Long-term storage	SPREC code
**EDTA**	BLD 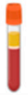	PED	**B1**: 2-10ºC<br><30 min	**B**: RT<br>10-15 min<br><3000g with braking	**J**: 2-10ºC<br>10-15 min<br>>10000g with braking	**A**: 2-10ºC<br><1h	**A**: PP 0.5-2 mL<br>(-85) to (-60)ºC 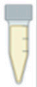	BLD-PED-B1-B-J-A-A
**ST-1**	BLD 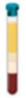	SCK	**E**: RT 4-8ºC	**B**: RT<br>10-15 min<br><3000g with braking	**N**: no centrifugation	**A**: 2-10ºC<br><1h	**A**: PP 0.5-2 mL<br>(-85) to (-60)ºC 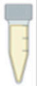	BLD-SCK-E-B-N-A-A
**ST-2**	BLD 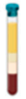	SCK	**E**: RT 4-8ºC	**B**: RT<br>10-15 min<br><3000g with braking	**J**: 2-10ºC<br>10-15 min<br>>10000g with braking	**A**: 2-10ºC<br><1h	**A**: PP 0.5-2 mL<br>(-85) to (-60)ºC 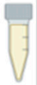	BLD-SCK-E-B-J-A-A

Figure S1. Summary of the procedures followed and the type of samples obtained. SPREC codes were used for reporting biospecimen handling. Created with BioRender.com. *BLD: Blood (whole); PED: Potassium EDTA; SCK: Nonaldehyde-based stabilizer for cell-free nucleic acids; PP: polypropylene.*

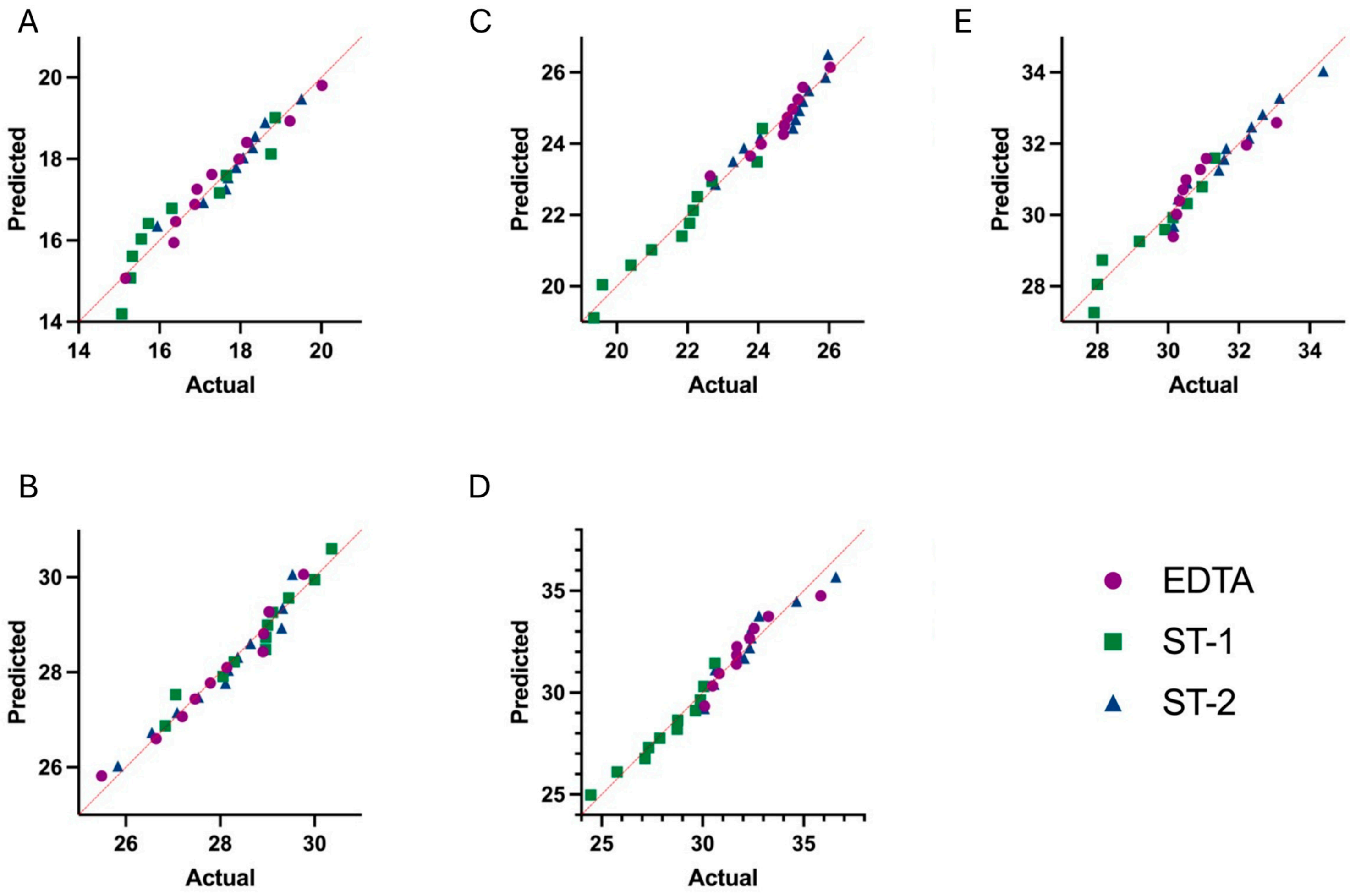

Figure S2. Quantile-Quantile plots using Cq values from miR-16 (A), miR-21 (B), miR-125b (C), miR-375 (D) and miR-182 (E) among conditions. Normality was assessed using Kolmogorov-Smirnov test and P-values were summarized on Table S1. Based on Cq values, all miRNAs follow a Gaussian distribution in all conditions, thus parametric test were used for their analysis.

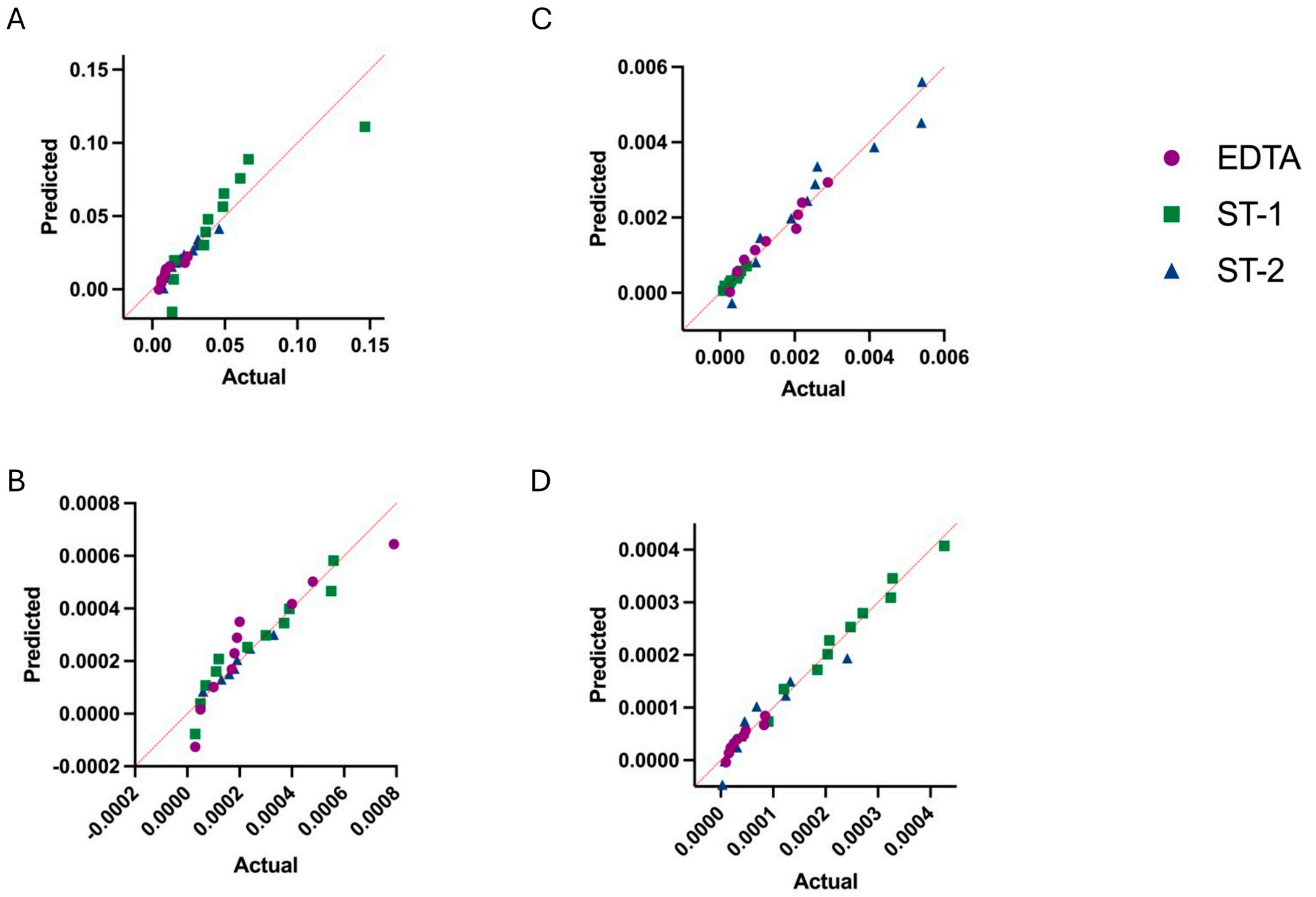

Figure S3. Quantile-Quantile plots using $2^{-\Delta Cq}$ values from miR-21 (A), miR-125b (B), miR-375 (C) and miR-182 (D) among conditions. Normality was assessed using Kolmogorov-Smirnov test and P-values were summarized on Table S1. Based on $2^{-\Delta Cq}$ values, not all miRNAs follow a Gaussian distribution in all conditions, thus non-parametric tests were used for their analysis.

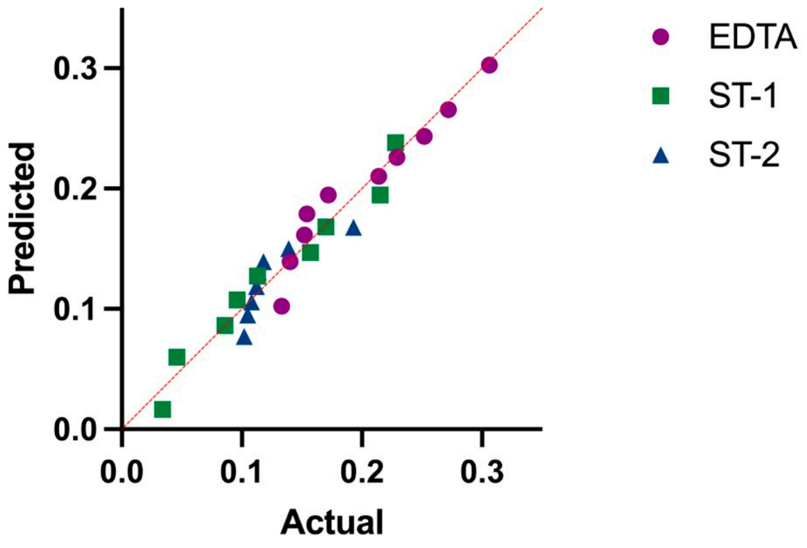

Figure S4. Quantile-Quantile plots using OD values from the three different conditions. Normality was assessed using Kolmogorov-Smirnov test and P-values were summarized on Table S1. Based on OD values, only ST-2 conditions did not follow a Gaussian distribution, thus non-parametric tests were used when considering this condition.

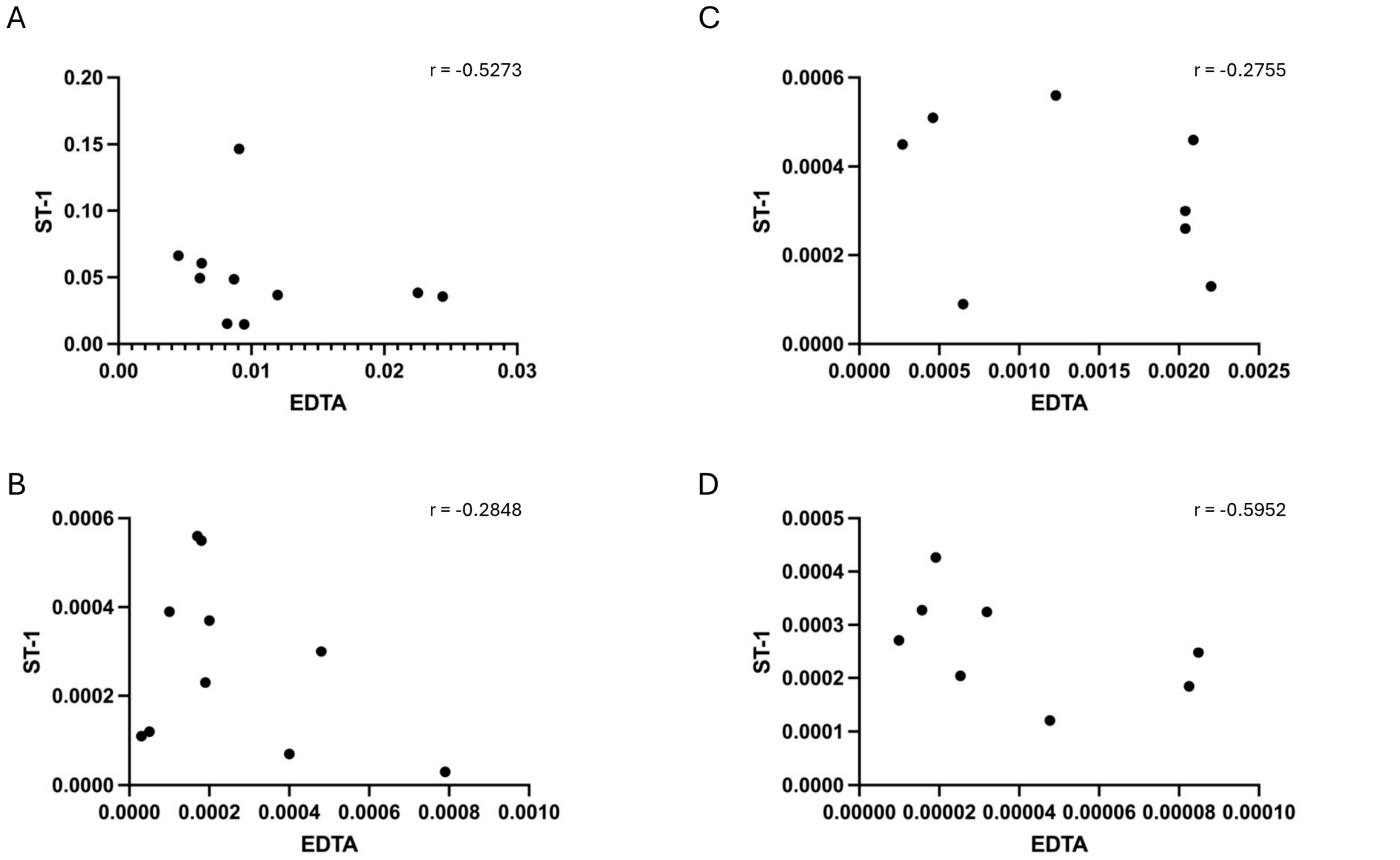

Figure S5. Correlation, using $2^{-\Delta Cq}$ values from miR-21 (A), miR-125b (B), miR-375 (C) and miR-182 (D), between EDTA and ST-1 conditions. Correlation was assessed using non-parametric Spearman tests and P-value was non-significant in any condition, when alpha=0.05. r = Spearman r.

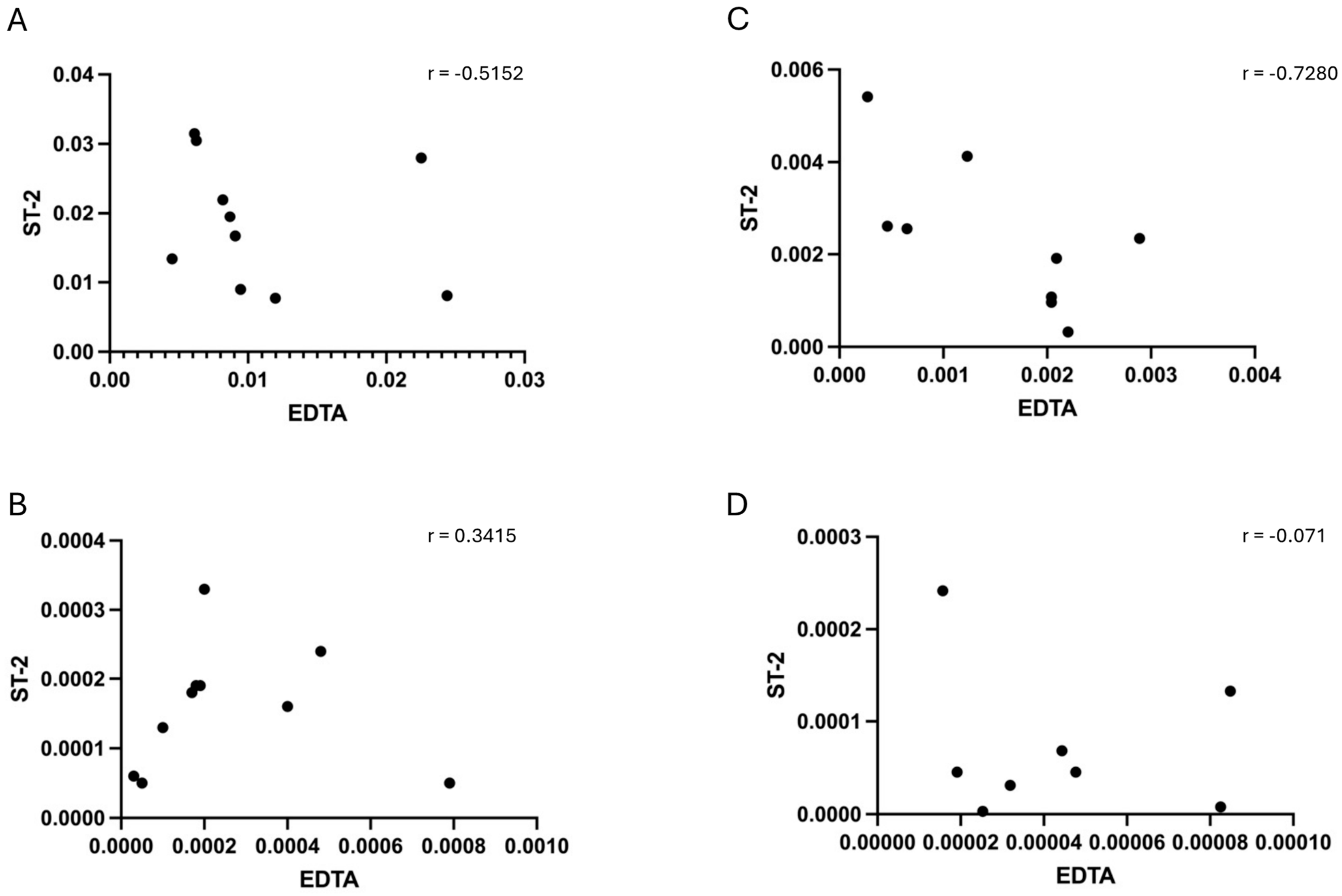

Figure S6. Correlation, using $2^{-\Delta Cq}$ values from miR-21 (A), miR-125b (B), miR-375 (C) and miR-182 (D), between EDTA and ST-2 conditions. Correlation was assessed using non-parametric Spearman tests and P-values was non-significant in any condition, when alpha = 0.05. r = Spearman r

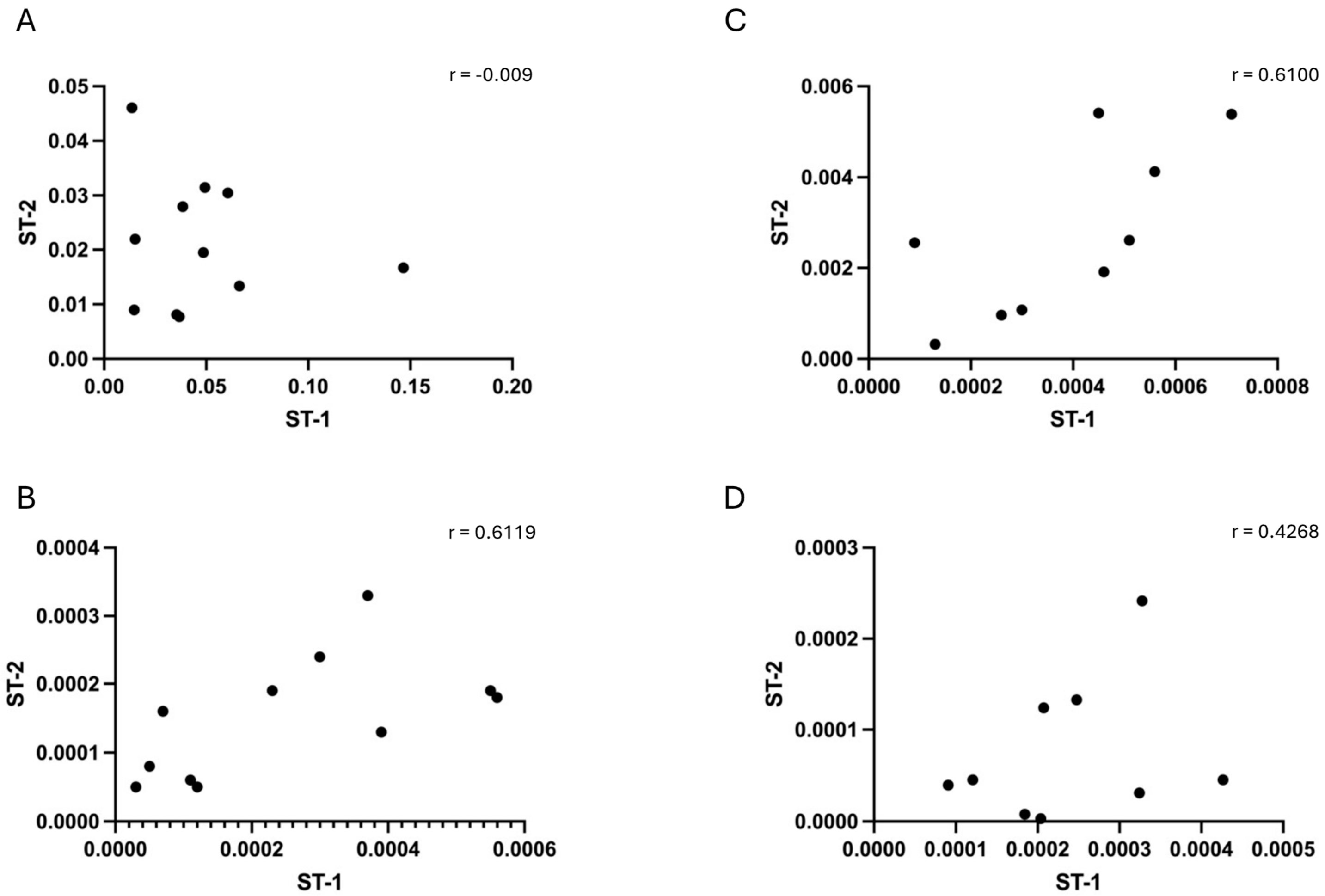

Figure S7. Correlation, using $2^{-\Delta Cq}$ values from miR-21 (A), miR-125b (B), miR-375 (C) and miR-182 (D), between EDTA and ST-2 conditions. Correlation was assessed using non-parametric Spearman tests and P-values was non-significant in any condition, when alpha = 0.05. r = Spearman r.
