# Supplementary material for: The Impact of Tube Type, Centrifugation Conditions, and Hemolysis on Plasma Circulating MicroRNAs
Source: Diagnostics (Basel). 2024 Oct 24;14(21):2369. doi: 10.3390/diagnostics14212369 (PMC11545111; doi:10.3390/diagnostics14212369)
Supplement: Supplementary file 1 [file diagnostics-14-02369-s001.zip › Table S1.pdf]

Table S1. Kolmogorov-Smirnov test results for each study population. The test is considered significant at an alpha level of 0.05.

		Kolmogorov-Smirnov test	
		P value	Normal distribution
miR-16 Cq	EDTA	>0.10000	Yes
	ST-1	>0.10000	Yes
	ST-2	>0.10000	Yes
miR-21 Cq	EDTA	>0.10000	Yes
	ST-1	>0.10000	Yes
	ST-2	>0.10000	Yes
miR-125b	EDTA	>0.10000	Yes
	ST-1	>0.10000	Yes
	ST-2	>0.10000	Yes
miR-375	EDTA	>0.10000	Yes
	ST-1	>0.10000	Yes
	ST-2	>0.10000	Yes
miR-182	EDTA	>0.10000	Yes
	ST-1	>0.10000	Yes
	ST-2	>0.10000	Yes
2^- ΔCqmiR21	EDTA	0.0130	No
	ST-1	>0.10000	Yes
	ST-2	>0.10000	Yes
2^- ΔCqmiR125b	EDTA	0.0114	No
	ST-1	>0.10000	Yes
	ST-2	>0.10000	Yes
2^- ΔCqmiR375	EDTA	>0.10000	Yes
	ST-1	>0.10000	Yes
	ST-2	>0.10000	Yes
2^- ΔCqmiR182	EDTA	0.0728	No
	ST-1	>0.10000	Yes
	ST-2	>0.10000	Yes
OD	EDTA	>0.10000	Yes
	ST-1	>0.10000	Yes
	ST-2	0.0033	No
